# Mechanisms of *Pseudomonas aeruginosa* Resilience Against Antibiotic Treatment and Outlooks of Emerging Treatment Strategies

**DOI:** 10.3390/medicina62010163

**Published:** 2026-01-13

**Authors:** Angelika Krūmiņa, Indra Zeltiņa, Paula Simsone, Emile Eulitz, Aigars Reinis, Ludmila Vīksna

**Affiliations:** 1 Department of Infectology, Riga Stradins University, 1007 Riga, Latvia; indra.zeltina@rsu.lv (I.Z.); paulasimsone@gmail.com (P.S.); ludmila.viksna@rsu.lv (L.V.); 2 Riga East Clinical University Hospital, 1079 Riga, Latvia; 3 Faculty of Medicine, Riga Stradins University, 1007 Riga, Latvia; 046256@rsu.edu.lv; 4 Department of Biology and Microbiology, Riga Stradins University, 1007 Riga, Latvia; aigars.reinis@rsu.lv; 5 Pauls Stradins Clinical University Hospital, 1002 Riga, Latvia

**Keywords:** *Pseudomonas aeruginosa*, difficult-to-treat resistant (DTR) *P. aeruginosa*, multidrug resistant *P. aeruginosa*, non-antibiotic therapies, emerging treatment, combination therapy, novel antibiotics

## Abstract

*Pseudomonas aeruginosa* is a resilient Gram-negative pathogen frequently implicated in healthcare associated infections, particularly among immunocompromised individuals and those with chronic conditions such as cystic fibrosis (CF), chronic obstructive pulmonary disease (COPD), or cancer. It is well known for its high resistance to antibiotic treatment. This review briefly mentions *P. aeruginosa’s* resistance mechanisms, biofilm formation, and virulence factors, while primarily focusing on treatment challenges and recent advancements in therapeutic strategies aimed at overcoming resistance. Covered are novel non-antibiotic interventions such as quorum sensing inhibitors, quorum quenching agents, iron chelators, lectin and efflux pump inhibitors, as well as antimicrobial peptides and nanoparticles. Traditional medicine, phytochemicals, and probiotics are also evaluated. Additionally, this review explores the development of a viable vaccine, bacteriophage therapy, lactoferrin-hypothiocyanite combination, and topical use of electrochemical scaffolds. This review emphasizes the need for extensive safety studies and in vivo validation of these emerging non-antibiotic therapeutic strategies to determine their efficacy, pharmacological behavior, and clinical feasibility before they can be translated into practice. Many of these emerging treatments could play a vital role in future combination therapies by enhancing the efficacy of existing antibiotics and countering resistance and virulence mechanisms. Advancing these approaches from laboratory to clinical application remains a major challenge, making the development of approved therapies or vaccines a critical scientific and public health priority.

## 1. Introduction

*Pseudomonas aeruginosa* is a significant cause of healthcare associated infections, especially in intensive care units [1]. It is considered an opportunistic pathogen, rarely causing disease in healthy individuals, but posing a serious threat to immunocompromised patients, including those with cystic fibrosis (CF), HIV/AIDS, malignancies, ocular or burn injuries, chronic non-healing diabetic wounds or indwelling medical devices [2,3,4,5,6]. Infections caused by *P. aeruginosa* in individuals with CF, healthcare-associated pneumonia, or chronic obstructive pulmonary disease (COPD) are associated with increased morbidity and mortality [5,7,8,9].

The resilience of *P. aeruginosa* against treatment stems from a plethora of resistance mechanisms—intrinsic, acquired and adaptive resistance [10,11,12]. Especially problematic for successful treatment is its ability to form biofilms—colonies of bacteria embedded in self-produced extracellular matrix. *P. aeruginosa* commonly forms these biofilms on indwelling catheters or within the airways of CF patients [13,14,15]. These structures hold a slow-growing subpopulation of the bacterium that possess high resistance to antibiotic treatment and is therefore very challenging to treat [13,16].

This literature review gives a brief insight into the resistance mechanisms and virulence factors of *P. aeruginosa*, with the focus on the emerging therapeutic approaches that have been proposed in recent years to combat antimicrobial resistance.

## 2. Challenges and Current Approaches in Managing *P. aeruginosa* Infections

*P. aeruginosa* is classified as an ESKAPE pathogen, a group of bacteria notable for “escaping” the effects of conventional antibiotics and causing severe hospital-acquired infections [17,18,19]. It primarily affects immunocompromised patients, such as those with CF, severe burns, or requiring prolonged mechanical ventilation [1,2,3,4,5,6].

*P. aeruginosa* thrives in moist environments and on medical devices like ventilators, contributing to its prevalence in intensive care units (ICUs) where it can cause pneumonia, urinary tract and bloodstream infections [1,3,20]. Its high mutation rate and adaptability allow it to rapidly develop antibiotic resistance, which is worsened by the selective pressure from widespread antibiotic use, making infections increasingly difficult to treat [16,17,18,21,22,23].

Conservative treatment of *P. aeruginosa* is challenging due to its vast collection of resistance mechanisms [24]. The pathogen can withstand a wide range of antibiotic compounds, including carbapenems, which are typically considered last-resort treatment options [25,26]. Also, β-lactam antibiotics, such as penicillin and cephalosporins, are often ineffective against *P. aeruginosa* due to production of AmpC β-lactamase [27,28].

Among newer treatment options, ceftolozane-tazobactam was found to have a reduced susceptibility to common resistance mechanisms of *P. aeruginosa*, such as efflux pump overexpression and decreased membrane permeability, compared to ceftazidime-avibactam, likely due to structural modifications that enhance stability and permeability [29,30]. Similarly, imipenem/relebactam and cefiderocol are mostly unaffected by resistance mechanisms in *P. aeruginosa*, such as efflux pump overexpression and decreased membrane permeability, maintaining potent in vitro activity where other β-lactams may fail [31,32]. Interestingly, strains resistant to ceftazidime-avibactam or ceftolozane-tazobactam may still be treatable with imipenem/relebactam or cefiderocol [33].

Multidrug-resistant (MDR) *P. aeruginosa* is defined as non-susceptibility to at least one agent in three or more antibiotic classes with expected antipseudomonal activity, whereas difficult-to-treat resistance (DTR) refers to *P. aeruginosa* that is non-susceptible to all standard first-line antipseudomonal agents, including piperacillin-tazobactam, ceftazidime, cefepime, aztreonam, carbapenems, and fluoroquinolones [34].

Current IDSA and ESCMID guidelines emphasize that the newer β-lactam/β-lactamase inhibitor combinations—ceftolozane/tazobactam; ceftazidime/avibactam; imipenem/relebactam; cefiderocol—should be reserved for infections caused by DTR *P. aeruginosa*, where no alternative effective agents are available. Their strategic use aligns with antimicrobial stewardship principles, aiming to preserve efficacy, limit the emergence of resistance, and optimize clinical outcomes [34,35].

## 3. Resistance Mechanisms

*P. aeruginosa* exhibits notable antibiotic resistance through three primary mechanisms: intrinsic, adaptive, and acquired resistance [10,11,12].

### 3.1. Intrinsic Resistance

*P. aeruginosa* exhibits intrinsic antibiotic resistance due to its outer membrane’s extremely low permeability—up to 100 times lower than *Escherichia coli*—which limits drug entry through porin channels [10,36]. Resistance is further enhanced by efflux pumps, which expel a broad range of antibiotics. Together with low membrane permeability and efflux pumps, these mechanisms form a robust intrinsic defense against antibiotic therapy [10,37,38].

In *P. aeruginosa*, efflux pumps belong mainly to the Resistance-Nodulation-Division (RND) family, including prominent systems such as MexAB-OprM, MexxXY-OprM, MexCD-OprJ, and MexEF-OprN [39,40]. These pumps can actively export multiple classes of antibiotics, including β-lactams, fluoroquinolones, macrolides, tetracyclines, chloramphenicol, novobiocin, reducing intracellular drug concentrations to sublethal levels [39,41,42]. The constitutive expression of some efflux systems, such as MexAB-OprM, contributes to baseline intrinsic resistance, while other RND efflux pumps (e.g., MexCD-OprJ and MexEF-OprN) are inducible but still part of the bacterium’s chromosomally encoded defense arsenal [39,40].

In addition to these physical barriers, *P. aeruginosa* naturally produces chromosomally encoded β-lactamases, such as OXA-50 and AmpC, which hydrolyze many β-lactam antibiotics and contribute to baseline resistance [10,43]. The combination of low membrane permeability, constitutive efflux pump activity, and endogenous β-lactamase production establishes a multi-layered intrinsic defense, making *P. aeruginosa* inherently less susceptible to a broad spectrum of antimicrobial agents [10,44].

### 3.2. Adaptive Resistance

Adaptive resistance in *P. aeruginosa* is a temporary, reversible response to environmental stressors, including exposure to antibiotics, oxidative stress, nutrient limitation, or hypoxia [10,45,46,47]. These stressors induce transient gene expression changes, enhancing defensive mechanisms such as efflux pump activity, porin downregulation, and AmpC-mediated-β-lactamase induction [10,45,46,47].

A key example of adaptive resistance is the induction of MexXY-OprM efflux pumps upon exposure to aminoglycosides, which transiently increases antibiotic tolerance [48].

Biofilm formation further amplifies adaptive resistance by creating localized gradients of antibiotics and stress, enhancing efflux activity and reducing drug penetration, which can result in antibiotic tolerance up to 1000-fold higher than planktonic cells [49]. Importantly, these mechanisms and non-mutational and reversible, distinguishing adaptive resistance from acquired resistance, and they enable *P. aeruginosa* to survive transient antimicrobial or environmental pressures [50].

### 3.3. Acquired Resistance

Acquired antibiotic resistance in *P. aeruginosa* is primarily mediated through horizontal gene transfer (HGT) and spontaneous mutations [10,22].

HGT allows the bacterium to acquire resistance determinants from other microorganisms via three main processes: conjugation, in which plasmids are directly transferred between cells; transformation, involving uptake of free DNA from the environment; and transduction, where bacteriophages deliver genes into recipient cells [51]. For instance, genes encoding β-lactamases, which hydrolyze β-lactam antibiotics, can be acquired through any of these HGT pathways [10,52]. Similarly, genes encoding aminoglycoside-modifying enzymes, which chemically inactivate aminoglycosides, may also be transferred via conjugation, transformation, or transduction [53].

Spontaneous mutations can confer stable, heritable resistance by modifying antibiotic targets, regulation efflux pumps, or altering porin channel permeability [22]. A well-documented example of mutation-driven resistance in *P. aeruginosa* is mutational inactivation of the OprD porin, leading to carbapenem, especially to imipenem, resistance [54].

## 4. Virulence Factors

*P. aeruginosa* have numerous virulence factors that allow it to survive in diverse environments and cause infections [3,12]. These virulence factors allow it to survive in diverse environments and cause infections. Quorum sensing (QS) allows groups of *P. aeruginosa* to sense population density and, in response to changing cell densities, coordinate the expression of numerous virulence factors, including surface structures such as flagella, pili and adhesins, as well as extracellular factors, including exotoxins (ExoS, ExoU, exotoxin A), proteases, elastases, rhamnolipids, siderophores, and Type II, III and VI secretion systems [3,10,18,55,56]. Many of these factors are packaged into extracellular vesicles, which facilitate bacterial survival, enhance virulence, and modulate host immune responses, particularly in challenging environments such as the lungs of cystic fibrosis patients [57]. Clinical isolates from CF lungs show vesicle-associated proteases and other components that interact with host cells and influence biofilm architecture, implicating OMVs as adaptive virulence elements [58]. Understanding these mechanisms is very important for developing new therapy approaches.

The most significant and commonly mentioned virulence factors of *P. aeruginosa* include lipopolysaccharides (LPS), which contribute to immune evasion and protection against antimicrobial peptides; outer membrane proteins and lipoproteins, which facilitate adhesion, nutrient uptake and interaction with host cells; biofilm formation, which provides structural protection and enhances antibiotic resistance; motility elements such as flagella and pili, which allow colonization and surface attachment; protein secretion systems (Types II, III, VI), which deliver toxins and enzymes directly into host cells; cell-to-cell communication through quorum sensing, which coordinates expression of virulence genes in population-density-dependent manner; and iron acquisition mechanisms, including siderophore-mediated chelation, which enable bacterial growth in iron-limited environments such as the human host [3,10,14,56,57,59,60].

Biofilm formation is one of the most important virulence and resistance strategies of *P. aeruginosa*, providing a protective matrix that shields bacterial communities from host immune defenses, antimicrobial agents, and environmental stresses [61,62]. Within biofilms, bacteria are embedded in an extracellular polymeric substance (EPS), composed of polysaccharides, proteins, lipids, and extracellular DNA, which not only enhances adhesion to surfaces but also facilitates intercellular communication, nutrient retention, and coordinated expression of virulence factors, ultimately contributing to chronic and persistent infections, particularly in immunocompromised patients [3,10,18,20,61,62].

## 5. Novel Treatment Strategies

Since their introduction in the early twentieth century, antibiotics have served as the principal modality for treating bacterial infections, revolutionizing modern medicine and saving millions of lives [63]. However, the widespread emergence of multidrug-resistant *P. aeruginosa* (MRPA) has increasingly compromised the efficacy of these conventional therapies, highlighting the urgent need for alternative treatment strategies [64]. This section therefore examines emerging therapeutic approaches that may offer viable alternatives to traditional antibiotic therapy.

### 5.1. Quorum-Sensing Inhibitors as a Non-Antibiotic Treatment Option

*P. aeruginosa* cell-to-cell communication mechanism also known as the quorum sensing (QS) system plays an irreplaceable role in the coordination, virulence and adaptability of the bacterium [3,10,18,55,56,65].

Quorum-sensing inhibitors (QSIs) represent a well-studied alternative therapeutic approach that functions by targeting *P. aeruginosa* virulence rather than their survival [66]. QSIs typically block quorum sensing either by preventing signal molecules from binding to their cognate regulators or by interfering with signal production, thereby disrupting communication and reducing expression of virulence factors such as biofilm formation and toxin production without directly affecting growth [66,67,68].

Several QSIs have been identified from a variety of natural sources, including bacteria, algae, and plants. The first anti-QS compounds were isolated from the red marine algae *Delisea pulchra* [69]. Subsequent studies identified similar QS inhibitors in brown algae such as *Ascophyllum nodosum* [70]. Plant extracts from species like *Syzygium aromaticum*, *Dalbergia thiocarpa*, *Terminalia chebula*, *Conocarpus erectus*, *Moringa oleifera* and macrolide antibiotic azithromycin have also demonstrated notable anti-quorum sensing activity [71,72,73,74,75].

Derived from the previously mentioned natural sources, compounds such as brominated furanones, 6-gingerol, eugenol, ajoene, and cos-2-dodecenoic acid have been identified and synthesized [76,77,78,79]. These compounds have been shown, both in vitro and some in vivo studies to inhibit quorum sensing-regulated virulence factors in *P. aeruginosa*, including proteases, pyoverdine, swarming motility, and biofilm formation [76,77,78,79].

Additionally, combination therapies using QSIs together with antibiotics have been shown to enhance bacterial susceptibility, reduce biofilm formation, and improve pathogen clearance compared to antibiotic monotherapy, with synergistic effects demonstrated in vitro and in vivo models. Furthermore, combining quorum quenching enzymes with bacteriophage treatment has been reported to increase phage susceptibility, supporting the potential of integrated anti-virulence strategies [80,81,82]. For instance, the combination of the QSI with tobramycin significantly enhanced antibacterial efficacy against *P. aeruginosa* biofilms, leading to greater reduction in bacterial load compared to antibiotic monotherapy in both in vitro and in vivo models [81].

Despite the promising potential of QSIs, several critical challenges must be addressed to enable their effective clinical application. A significant challenge in the development of QSIs lie in their delivery and bioavailability, as many compounds fail to achieve therapeutic concentrations at the infection site due to poor absorption, rapid degradation, or limited tissue penetration. This can compromise their clinical efficacy and complicate dose optimization. Host safety also remains a concern, since certain QSIs may inadvertently impact human cells or disturb the normal microbiota, resulting in adverse effects. Moreover, although QSIs generally exert lower selective pressure than conventional bactericidal agents, there is still a potential for the emergence of quorum sensing-resistant pathogens over time, which could diminish their long-term effectiveness. These considerations underscore the importance of optimized formulations, precise delivery strategies, and ongoing surveillance to enable the safe and effective clinical application of QSIs [83,84,85].

### 5.2. Iron Chelators

Iron is a fundamental necessity for *P. aeruginosa* growth and function, as it supports the proper activity of key iron-dependent enzymes such as ribonucleotide reductase, respiratory cytochrome complexes, succinate dehydrogenase, NADH dehydrogenase, aconitase as well as oxidative-stress-response enzymes including catalases and Fe-superoxide dismutase, which collectively underpin DNA and protein synthesis, electron transport, and protection against oxidative damage [86,87]. To meet this demand in iron-limited host environments, *P. aeruginosa* relies primarily on two well-characterized iron acquisition systems: the high-affinity pyoverdine system and the lower-affinity pyochelin system [86,87]. These siderophores chelate extracellular ferric iron (Fe^3+^) and mediate its transport into the cell. Pyoverdine production and secretion are tightly regulated by the extracytoplasmatic function (ECF) σ factor PvdS, whose expression is controlled by iron availability through the ferric uptake regulator Fur [86,87]. Binding of pyoverdine to its outer membrane receptor initiates a signaling cascade that modulates PvdS activity, thereby coordinating pyoverdine biosynthesis with the expression of iron-responsive genes, including virulence factors such as exotoxin A [86,87]

Host defenses, such as lactoferrin, limit iron availability, inducing bacterial twitching, preventing microcolony formation, reducing virulence, and enhancing antibiotic susceptibility [88,89]. Exploiting this dependence, therapeutic strategies using iron chelators have shown promise: gallium compounds mimic Fe^3+^ impairing siderophore-mediated uptake [90,91]; hydroxamates (deferoxamine) [90], aminocarboxylates (EDTA, DTPA) [90,92], and hydroxypyridiones (DFP, CPX, DIBI) [90,93] chelate iron and inhibit biofilm growth [90]; combination therapies with antibiotics enhance efficacy against biofilm-embedded bacteria.

Studies show that combining chelators with antibiotics—e.g., DFO-ga with gentamicin, desferrioxamine or deferasirox with tobramycin, or gallium, colistin, and surfactants targeting central biofilm cells enhances antimicrobial efficacy compared to individual agents alone [90,94,95].

Iron chelation based anti-virulence strategies show promise, yet several key limitations hinder their clinical translation. Cytotoxicity and host safety remain significant concerns, as approved chelators can induce systemic adverse effects. Their antimicrobial activity is also highly strain-dependent, varying with bacterial iron-uptake pathways, metabolic state and environmental conditions. Poor stability, limited tissue penetration, and rapid physiological inactivation further reduce their ability to achieve effective iron sequestration in vivo. Additionally, iron deprivation may induce compensatory bacterial responses, including enhanced siderophore production or altered quorum-sensing activity, while biofilm-associated iron stores and polymicrobial competition can diminish therapeutic impact. These limitations highlight the need for optimized formulations, targeted delivery approaches, and robust in vivo validation before clinical application [96,97].

### 5.3. Inhibition of Lectins in P. aeruginosa

Lectins are carbohydrate-binding proteins that contribute significantly to the bacterium’s virulence [98]. They enable the bacteria to adhere to host’s tissues, initiate and sustain biofilm formation, and evade immune responses. By specifically recognizing and binding to glucose residues on host cell surfaces or within the extracellular matrix, lectins support colonization, persistence, and the successful proliferation of an infection [99,100].

The principle behind lectin inhibition is to prevent lectins from binding to host cells. Lectin inhibitors are also considered to be more stable than other compounds and carry a low risk of bacteria developing resistance against them [101].

In *P. aeruginosa* adhesion to lung epithelial cells is primarily mediated by the lectins LecA and LecB, which bind specifically to galactose- and fuctose-containing receptors on the epithelial surface [101,102]. This interaction promotes bacterial attachment and disrupts ciliary beating, thereby enhancing virulence [102,103]. Lectin inhibition aims to block lectin-host cell interactions and is considered a stable anti-adhesion strategy with a low risk of resistance development [99].

In a 2011 study, the β-phenylgalactosyl peptide dendrimer (GalAG2), a glycopeptide dendrimer, demonstrated strong binding affinity to *P. aeruginosa* LecA and effectively inhibited biofilm formation in vitro. The multivalent design of GalAG2 not only dramatically increased its binding affinity to LecA compared with simple monovalent galactosides but also enabled complete inhibition and even dispersal of established *P. aeruginosa* biofilms at micromolar concentrations, highlighting the importance of multivalency for functional antivirulence activity [104].

Recently, dual inhibitors targeting both the lectin LecA and the protease LasB have been proposed [105]. Using a molecular hybridization approach, researchers developed a potent, selective and non-toxic thiol-based inhibitor that simultaneously blocks the two major extracellular virulence factors of *P. aeruginosa*. This synergistically reduces the pathogen’s virulence. The bifunctional LecA/LasB inhibitor (named compound 12) demonstrated superior efficacy in inhibiting virulence compared to individual inhibitors or combinations of individual inhibitors in vitro. The study suggested that dual inhibitors offer even more effective treatment option for *P. aeruginosa* infections and that this approach could be extended to other targets and perhaps even other pathogens if required [105].

Although the results of lectin inhibitor research are promising and already quite advanced, the design complexity of these compounds may pose potential side effects or other yet-unknown medical concerns. Most high-affinity lectin antagonists rely on multivalent presentations that lead to large, complex molecules with unfavorable pharmacokinetic profiles and potential off-target interactions. Many of these inhibitors are also difficult and costly to synthesize, and there is a lack of robust in vivo and clinical data confirming their safety and efficacy. In addition, lectin inhibition efficacy can be strain-dependent, which may limit broad applicability. Before clinical trials can begin, extensive in vitro and in vivo animal testing will be necessary to ensure the safety and effectiveness of newly engineered compounds. Despite these challenges and the time required for development, synthetic lectin inhibitors still show great potential to transform approaches to disrupting *P. aeruginosa* biofilms [106,107].

### 5.4. Inhibition of Efflux Pumps in P. aeruginosa

Efflux pumps in *P. aeruginosa* play a crucial regulatory role, which contributes to increased antibiotic resistance through the active expulsion of antimicrobial agents and to the secretion of virulence factors [108]. Therefore, the possibility of inhibiting these important structures is considered a valuable approach to dampen the virulence of *P. aeruginosa* as well as reduce its antibiotic resistance [108].

A well-known example of efflux pump inhibitors (EPI) is phenylarginine-β-naphthylamide (PAβN), a synthetic peptidomimetic compound designed to inhibit RND family efflux pumps, such as MexAB-OprM and MexXY-OprM, which are important contributors to *P. aeruginosa’s* intrinsic and acquired multidrug resistance [109].

Interestingly, the MexAB-OprM efflux pump is overexpressed under severe iron limitation, highlighting its role in *P. aeruginosa* survival [110]. Accordingly, combined treatment with iron chelators and the efflux pump inhibitor PaβN acts synergistically to enhance growth inhibition, impair biofilm formation and increase *P. aeruginosa* susceptibility to ciprofloxacin in vitro [110].

A recent strategy combines PaβN with antimicrobial photodynamic inactivation (APDI), in which methylene blue (MB) penetrates microbial cells and binds to membranes, DNA, and proteins. Upon visible light exposure, excited MB transfers energy to oxygen, generating intracellular ROS that induce oxidative stress and cause cellular and DNA damage [111,112]. The clinical application of ADPI is limited in conditions such as CF, bacteremia, and urinary tract infections due to its dependence on light activation, nonetheless, future development of fiber optic based devices could enable targeted illumination of otherwise inaccessible biofilms [113].

In addition to novel methods like APDI, another promising approach is the repurposing of approved drugs as potential efflux pump inhibitors. A 2024 study evaluated promethazine (PMZ), fluoxetine (FLU), and carbonyl cyanide 3-chlorophenylhydrazone (CCCP) as EPIs MRPA [114]. Subinhibitory concentrations increased ethidium bromide accumulation, confirming efflux inhibition and reduced metabolic activity and biomass of mature biofilms. Although, the effective concentrations exceeded clinically achievable levels, these compounds lowered antimicrobial MICs in both planktonic cells and biofilms, supporting the role of efflux pumps in antibiotic resistance. Based on these findings, the newly characterized EPIs should be incorporated to broaden the range of compounds available for research, including potential application in long-term indwelling medical devices [114,115]

Despite the promising potential of EPIs to combat MRPA, their development and clinical application face several significant challenges. Many candidate EPIs exhibit potent activity in vitro but fail to maintain efficacy in complex biological systems due to structural versatility and broad substrate range of efflux pumps, which complicates the prediction of inhibitor-pump interactions. Additionally, the large chemically complex structures required for effective inhibition often result in poor pharmacokinetic properties, low solubility, and limited tissue penetration, while some compounds display toxicity or off-target effects. Effective concentrations frequently exceed clinically achievable levels, and combining EPIs with antibiotics introduces further pharmacokinetic and safety considerations. Moreover, regulatory hurdles, high development costs, and the potential for resistance evolution against EPI-antibiotic combinations have so far prevented any EPI from reaching clinical approval. These limitations underscore the need for careful design, optimization, and in vivo evaluation to translate promising in vitro results into safe and effective therapies [116,117,118].

### 5.5. Traditional, Natural and Phytochemical Medications Against P. aeruginosa

#### 5.5.1. *Glycyrrhiza glabra* (Licorice root)

The natural flavoring agent *Glycyrrhiza glabra* demonstrated notable bactericidal activity against resistant strains of *P. aeruginosa*, even outperforming the control antibiotic, amikacin in vitro, suggesting its potential as an alternative antimicrobial agent [119]. This activity has been linked in other studies to the diverse bioactive phenolic compounds present in *G. glabra* extracts, which are known to contribute to antimicrobial effects through multiple mechanisms, including disruption of bacterial structures and redox-related pathways [120,121].

Although *G. glabra* exhibits promising antimicrobial activity in vitro, its clinical utility is limited by safety considerations and a lack of robust human data, as high doses or prolonged use may lead to adverse effects and long-term efficacy remains under-investigated [122].

#### 5.5.2. Ayurvedic Medicine and Herbal Formulations

Ayurvedic polyherbal formulations, widely used across Asian countries to treat infections and support immune system, have been reported to possess antimicrobial activity. Preparations such as *Triphala churna*, *Hareetaki churna* and *Dashmula churna* contain diverse medicinal plant extracts with demonstrated effects against *P. aeruginosa* under in vitro conditions [123]. Several studies also show that their phytochemicals can enhance the efficacy of conventional antibiotics, for instance, protocatechuic, ellagic and gallic acids enhance the activity of sulfamethoxazole or tetracycline, while allicin, a bioactive compound from garlic, can enhance the antibacterial activity of β-lactam antibiotics, including cefoperazone against *P. aeruginosa* under in vitro conditions [124,125].

Despite their promising antimicrobial properties, Ayurvedic polyherbal formulations face several challenges that limit clinical application. Variability in plant sources and preparation methods leads to inconsistent efficacy, while the complex mixture of bioactive compounds complicates dose optimization and safety assessment. Additionally, limited in vivo and toxicological data, potential drug-herb interactions, and insufficient clinical evidence hinder regulatory approval and broader adoption in modern medicine [126].

#### 5.5.3. Oils and Leaf Extracts

Leaf extracts from *Kalanchoe pinnata*, *Cynodon dactylon* and *Ocimum tenuiflorum* exhibit antimicrobial activity against *P. aeruginosa* and *S. aureus* [127]. When formulated in sesame oil, these preparations significantly reduced P. aeruginosa biofilm biomass and metabolic activity in vitro, and some formulations also inhibited planktonic growth. The sesame oil not only serves as a carrier but may enhance the penetration and stability of bioactive compounds, improving their efficacy. The antimicrobial activity is attributed to phytochemicals such as flavonoids, phenolic acids, and terpenoids, which can disrupt bacterial membranes, inhibit biofilm formation, and induce oxidative stress in pathogens. While these findings highlight the potential of these leaf extracts and oil-based formulations as alternative or complementary antimicrobial agents, further in vivo studies and clinical evaluations are required to confirm their safety and therapeutic utility [128].

#### 5.5.4. Phytochemical–Antibiotic Combinations

Phytochemical–antibiotic combinations have also been explored against MRPA strains. Extracts of *Rhus coriaria* seed, *Sarcopoterium spinosum* seed, and *Rosa damascena* flower were combined with selected antimicrobial agents such as oxytetracycline, penicillin G, cephalexin, sulfadimethoxine and enrofloxacin and decent results indicating antibacterial action were observed [124,128]. Additionally, medicinal plants like *Terminalia belilrica*, *Celastrus paniculatus*, *Kingiodendron pinnatum*, *Schleichera oleosa*, *Melastoma malabathricum*, and *Garcinia gummi-gutta* have also been subject to research for their activity against MRPA isolates [129,130]. Among them, the extract of *Terminalia bellerica* was found to be the most effective, significantly reducing pyocyanin production, exopolysaccharide synthesis, and biofilm formation in *P. aeruginosa* strains [130].

Although phytochemical-antibiotic combinations show promising in vitro synergy, their clinical use is limited by variability in plant extracts, inconsistent bioactive compound content and poor bioavailability. Potential herb-drug interactions, along with insufficient toxicological and clinical data, further hinder their translation into practice, emphasizing the need for rigorous preclinical and human studies [131].

### 5.6. Immunization Against P. aeruginosa Using Novel Vaccines

The primary goal of vaccine development is to prevent infection before it becomes established. Therefore, vaccines focus on preventing and reducing *P. aeruginosa* infections by counteracting its antigens, which induce a potent immune response and are responsible for pathogenesis [132]. While the idea of developing a vaccine against *P. aeruginosa* is certainly not new, despite extensive research, no licensed vaccine is currently available [132,133].

The 2018 review on *P. aeruginosa* vaccines proposed a framework for potentially effective immunization [134]. Murine infection models suggested that protection requires a combination of immune responses, including opsonophagocytic killing antibodies (OPK), antitoxin and anti-attachment antibodies, and T cell mediated immunity, particularly Th17 responses to address the bacterium’s diverse virulence [134,135]. However, a deeper understanding of human immune responses to *P. aeruginosa* remains necessary before progressing to clinical vaccination trials, though a future vaccine remains a feasible prospect [132,134].

The most recent publication on this matter is from February of 2025. It combines a valuable summary on *P. aeruginosa* resistance and virulence factors with the historical and recent progress on creating a vaccine [136]. Various vaccine strategies against *P. aeruginosa* have been explored, including outer membrane protein (OMP)-, LPS-, flagellin-, inactivated-, and alginate-based vaccines. OMPs and LPS elicit antibodies that prevent colonization and neutralize the pathogen [137], while flagellin can serve as both antigen and TLR5-stimulating adjuvant [138]. Inactivated vaccines are generally safe but require boosters due to weaker immunity [136], and alginate-targeting approaches aim to disrupt biofilm formation, particularly in CF patients [139]. Preclinical studies indicate that multivalent formulations combining OMPs and flagellin can enhance IgG responses, bacterial clearance, and reduce tissue damage [136].

Despite promising immunogenicity, challenges remain due to *P. aeruginosa* broad virulence, adaptability, and resistance mechanisms. Th17 responses and IgA induction are critical for mucosal protection, while safety concerns limit live-attenuated approaches. Emerging DNA and RNA vaccines offer multivalent flexibility and rapid design potential, suggesting that future breakthroughs may be achievable through combined strategies and advanced platforms [136].

### 5.7. Nanoparticles (NPs) Against P. aeruginosa

Nanoparticles (NPs) have recently attracted significant scientific interest for treating various diseases, including cancer and bacterial infections [140]. These tiny materials, typically less than 100 nm in size, have a high surface area-to-mass ratio and are already widely applied in chemical, biological, and biomedical fields [140,141]. In antimicrobial applications, nanoparticles are interesting because they can penetrate bacterial membranes, disrupt biofilm formation and serve as efficient carriers for antibiotics [142,143]. Additionally, nanoparticles have numerous antimicrobial mechanisms to their disposal, which make them an attractive new treatment option against *P. aeruginosa.*

Metallic nanoparticles have been widely investigated for their potential in preventing *P. aeruginosa* infections. Many metallic nanoparticles exhibit broad-spectrum antibacterial activity on their own [144]. Their small size enables them to penetrate bacterial membranes easily and exert antimicrobial effects. Various types such as zinc oxide, copper oxide, iron (III) oxide, titanium dioxide, lanthanum calcium manganate (LCMO), cerium oxide (CeO_2_), gold-functionalized magnetic nanoparticles, silver-gold alloys, dextrose-reduced gelatin-capped silver and nitric oxide-releasing silica nanoparticles have shown promising activity against *P. aeruginosa* [145,146,147].

Once the NPs attach to and penetrate the bacterial cell wall, several antimicrobial mechanisms are activated. Metallic NPs, such as those containing silver (Ag^+^), zinc (Zn^2+^), and copper (Cu^2+^), slowly release metal ions that disrupt enzymatic activity and essential cellular processes, as well as induce oxidative stress, leading to bacterial cell death. Additionally, NPs can enter the bacterial cytoplasm and interact with DNA or ribosomes, thus inhibiting replication and protein synthesis and effectively arresting bacterial growth [146,147,148].

Some of the most used inorganic NPs for antibacterial applications are based on silver, iron oxide, zinc oxide, titanium dioxide, magnesium oxide, and silica. These nanoparticles can also serve as drug delivery systems, with antibiotics either coated onto their surface or incorporated into their porous structure [149]. A wide range of antimicrobial agents such as streptomycin, neomycin, vancomycin, cephalexin, and ciprofloxacin have been successfully loaded onto NPs, enhancing their effectiveness against various bacterial pathogens. This is regarded to as “loading” the nanoparticles or “loaded NPs” [150,151,152]

These antibiotic-loaded inorganic NPs have demonstrated positive results in combating clinically relevant strains, including *Staphylococcus aureus*, *P. aeruginosa*, *Bacillus subtilis*, *Bacillus cereus*, *Streptococcus* species, *Escherichia coli*, and *Salmonella typhimurium* [153].

One of such loading approaches involves loading carvacrol, a natural phenol, into chitosan-based NP for the treatment of biofilm-associated infections. The study demonstrated that grafting an 11-carbon alkyl chain onto chitosan significantly enhanced its ability to penetrate the *P. aeruginosa* biofilms, effectively disrupting the biofilm structure and reducing the number of viable bacterial cells. The carvacrol–chitosan–SH11 nanoparticles altered the packing density of phospholipid monolayers, modifying compression isotherms and causing greater membrane expansion in the model systems. Furthermore, NPs incorporating carvacrol and chitosan with different alkyl chain lengths were able to inhibit both biofilm formation and bacterial motility [154].

A similar strategy involves incorporating NP into medical materials, such as wound dressings, to combat *P. aeruginosa* skin infections. Despite this progress, no clinical trials have yet been performed so the effectiveness of NP-loaded wound dressings in patients is hard to evaluate. This delay may stem from the lack of universally accepted safety standards for assessing the specific risks associated with NP use in clinical settings [155].

### 5.8. Bacteriophage Therapy

Bacteriophages are viruses that lyse bacterial, with lytic phages currently being the only type used clinically [156,157]. They infect bacterial cells, replicate and release progeny that continue targeting the same bacterial species [156,157]. Phage therapy offers several advantages, including high specificity, minimal disruption of normal microbiota, activity against antibiotic-resistant strains, and replication directly at the infection site [156].

As earlier presented, *P. aeruginosa* commonly forms biofilms on medical implants like catheters and in the airways of CF patients, making these infections highly resistant to antibiotics. However, bacteriophages can target these bacteria on the surface of biofilms and replicate locally. This increases their concentration at the infection site and promotes bacterial clearance [156]. Moreover, some phages are capable of producing alginase, an enzyme that breaks down the alginic acid component of the biofilm matrix, thereby disrupting the biofilm structure and hindering bacterial growth [156,157].

Bacteriophages have additional anti-biofilm mechanisms. They can inhibit biofilm formation by targeting the quorum sensing system of *P. aeruginosa* and therefore block bacterial communication, which is essential for biofilm development [156]. Also, phages can penetrate the biofilm of *P. aeruginosa* through the void spaces and channels. This enables them to reach the inner layers of the biofilm and start replicating without destroying the external matrix [156].

The anti-biofilm activity of phages can be supplemented through combination therapy with antibiotics [158]. Multiple reviewed studies have demonstrated that this type of combination therapy is much more effective than either treatment alone. For example, when *P. aeruginosa* was treated with a lytic bacteriophage alongside streptomycin or gentamicin, the combined therapy significantly reduced bacterial load compared to the effects of either agent used individually [159,160]. Similarly to the combination therapy with antibiotics, the phages can be combined with molecules that possess anti-biofilm properties such as enzymes or disinfectants [156].

Numerous in vitro and in vivo studies have evaluated phage effectiveness in combating chronic *P. aeruginosa* infections [116]. Based on these studies several phage prototypes have been developed and shown effective against *P. aeruginosa*, for example, E79, JG024, PaP1, MPK1, MPK6, PAK-P1, M4, LUZ7, and PIK. The effects of MPK1, MPK6, and PAK-P1 were tested in murine models, and it was found that intramuscular and intraperitoneal administration of MPK1 and MPK6 successfully protected mice from mortality due to *P. aeruginosa*-induced peritonitis and sepsis [161,162]. Additionally, PAK-P1 demonstrated strong protective effects by preventing lethal infections and effectively reducing lung colonization by *P. aeruginosa* in the animals [163]

Phages work by being able to recognize and attach to various molecules on the surface of bacteria, such as LPS, peptidoglycan, OMP, and teichoic acids. Therefore, the host range of a phage is determined by its receptor specificity. While some phages have a broad host range, others are limited to a narrow spectrum. Narrow host range phages can be ineffective, especially in polymicrobial biofilms formed on medical devices [156].

To address this challenge, the use of phage cocktails could be a viable solution for targeting polymicrobial biofilms [156]. Personalized cocktails are tailored to a patient’s specific bacterial strains, whereas fixed cocktails target a broader range of clinical isolates [164]. For instance, researchers under Forti et al. developed a cocktail containing six phages namely E215, E217, PAK_P1, PYO2, DEV, and PAK_P4, that demonstrated effectiveness against clinical *P. aeruginosa* strains [164].

Although the phage therapy approach is a strong prospect and has many redeeming properties, some problematic downsides have been revealed. For example, when phages were applied to burn wound infections, only minimal effects were observed, suggesting that some types of topical infections may not be able to be effectively treated with phages [165]. Other major challenges include the development of phage-neutralizing antibodies by the host immune system and safety concerns regarding exotoxins that can be present during phage production [166].

Another major limitation of phage therapy is the rapid emergence of bacterial resistance, partly driven by CRISPR-Cas system, which is an adaptive immune defense that allows bacteria to degrade invading phage DNA. During further research, researchers found protein AcrIF, a potent inhibitor of this system in *P. aeruginosa*. However, this protein is locked by part of the CRISPR system called Csy-complex (Type I–F Cascade complex), depriving phages of the ability to bind to the pathogen’s DNA. It was suggested to harness the same mechanisms by engineering phages who are then resistant to bacterial CRISPR defenses. As a solution, the development of synthetic phages with tailored characteristics and genomic content has been proposed for future research [167,168].

There are safety concerns related to phage clearance after treatment, the potential impurity of phage preparations, the poor stability of phage formulations, and a limited understanding of detailed mechanisms of action [169]. Pharmacodynamics and dosing considerations are also critical for effective therapy, as in vivo replication and distribution can vary depending on infection site and phage properties. Regulatory status is another important factor. Phage therapy is currently limited to experimental or compassionate-use frameworks in most countries, with clinical trials ongoing to evaluate safety and efficacy. Comprehensive guidelines are still being developed to ensure standardization, quality, and patient safety [166,169].

In conclusion, bacteriophage therapy represents a highly promising and modifiable alternative to traditional antibiotics in the fight against MRPA. Phages offer unique advantages, including specificity, self-replication at infection sites, strong anti-biofilm activity and the possibility to be combined with other treatment methods. However, despite these strengths, challenges such as bacterial resistance mechanisms, immune system neutralization, and safety concerns related to phage production must be overcome first. Advances like the development of phage cocktails, combination therapies, and synthetic phages can offer exciting potential, and personalized approaches further enhance their applicability. Further research as well as extensive clinical trials are necessary before phage therapy can be safely adopted in modern medical practice.

### 5.9. Antimicrobial Peptides as Antimicrobial Agents

Antimicrobial peptides (AMPs) have gained significant attention as potential antimicrobial agents in recent years. Produced by a variety of organisms, including bacteria, fungi, plants, invertebrates, and vertebrates, AMPs play a crucial role in their natural defense mechanisms [170]. These peptides target the membranes of bacteria, fungi, and protozoa, forming ion-channel pores that disrupt membrane integrity [170,171]. In addition, AMPs have been shown to possess anti-biofilm and immunomodulatory properties [172,173]. They exhibit rapid killing kinetics, low levels of induced resistance, and minimal toxicity to the host. And due to their broad-spectrum activity, AMPs could become a future alternative to conventional antibiotics to combat bacterial infections [172,173].

AMPs are highly diverse in terms of length (ranging from approximately 12 to 50 amino acids), sequence, and structure, yet most are small, cationic, and amphipathic [174]. Examples of AMPs include α-defensins (HNP-1 to HNP-4), cecropin P1, protegrin-1, LL-37, magainin2, indolicidin, thanatin, buforin II, and melittin. These peptides demonstrated broad-spectrum activity against various Gram-positive and Gram-negative bacteria, yeasts, fungi, and enveloped viruses [175].

De novo designed amphiphilic AMPs, such as G(IIKK)_3_I-NH_2_ (G3) and C8-G(IIKK)_2_I-NH_2_ (C8G2), have exhibited broad-spectrum antimicrobial activity during recent studies [134]. In Gram-negative bacteria, AMPs penetrate the outer membrane through self-promoted uptake pathway. The process begins with the peptide binding to surface LPS and displacing divalent cations that stabilize adjacent LPS molecules. This destabilizes the entire outer membrane, facilitating the uptake of more AMPs and leading to the formation of channels in the cytoplasmic membrane, which ultimately results in cell death [176].

Additionally, some AMPs have demonstrated synergistic properties together with conventional antibiotics against various bacteria, including *P. aeruginosa* [177,178]. This is due to the AMPs enhancing antibiotic uptake, disrupting biofilm formation and inhibiting bacterial quorum sensing [177]. In several trials, multiple combinations were tested. For example, GL13K with tobramycin improved clearance of *P. aeruginosa* biofilms [179]. Another research showed that the MIC of cecropin A2 decreased by 8-fold when combined with tetracycline antibiotics in vitro. This combination also had a positive effect during an in vivo trial with *Galleria mellonella* larvae, displaying an increased survivability [180].

Unfortunately, these antimicrobial peptides also present certain challenges and issues, including hemolytic activity toward host cells and reduced effectiveness due to sensitivity to salt, serum and pH variations. Additionally, there is risk of rapid degradation in the human body because of their susceptibility to proteolysis. Also, a high cost associated with production [181].

### 5.10. Development of New Antibiotic Formulations Against P. aeruginosa

Over the past decades, numerous novel non-antibiotic and antibiotic strategies have been explored against MRPA, often showing synergy with conventional antibiotics. While the development of new antibiotics remains critical, rising resistance limits their long-term efficacy, highlighting the need for innovative therapeutic approaches.

Among recent antibiotics, doripenem, a carbapenem inhibiting cell wall synthesis via penicillin-binding proteins, demonstrates enhanced in vitro activity against *P. aeruginosa* from CF patients and higher clinical cure rates in ventilator-associated pneumonia compared to imipenem, though side effects such as headache and gastrointestinal discomfort have been reported [182,183].

Plazmocin, a-next-generation aminoglycoside, inhibits bacterial protein synthesis and retains activity against most aminoglycoside-modifying enzymes, showing broad-spectrum efficacy against Gram-negative and Gram-positive pathogens, including multidrug-resistant strains [184,185,186]. Clinical data from phase III trials demonstrate its effectiveness in treating complicated urinary tract infections and bloodstream infections [185]. In addition to its standalone activity, plazmocin exhibits in vitro synergistic effects when combined with β-lactam antibiotics such as cefepime, doripenem, imipenem, or piperacillin-tazobactam, enhancing antibacterial activity against DTR *P. aeruginosa* and other resistant Gram-negative bacteria [186]. While generally well tolerated, plazmocin treatment can occasionally result in mild nephrotoxicity and ototoxicity [184,185,186].

Fluroquinolone delafloxacin targets DNA gyrase and topoisomerase IV, maintaining activity in acidic environments such as CF airways, while protein epitope mimetic (PEM) molecules like POL7001 inhibit LPS transport to the outer membrane, showing potent efficacy in vitro and in murine pneumonia models without reported toxicity [187,188]. Dual-mechanism agents such as zidebactam inhibit PBP2 and β-lactamases, extending activity against resistant Gram-negative bacteria, though clinical availability remains limited [189].

Overall, although progress has been made in developing new antibiotics, most represent refinements of existing drug classes rather than agents with truly novel mechanisms, limiting their long-term effectiveness against rapidly evolving pathogens. Only a small number show meaningful activity against most challenging organisms, such as *Acinetobacter baumanii* and MRPA, underscoring the need for genuinely innovative therapies. While continuous antibiotic development remains essential to stay ahead of resistance, addressing the underlying drivers through rigorous infection control and responsible antimicrobial use is even more critical.

### 5.11. Probiotics

As the search for new treatment strategies continues, probiotics have increasingly gained attention for their potential health benefits and over-the-counter availability. Probiotics are live, nonpathogenic microorganisms, used to enhance microbial balance, particularly in the gastrointestinal tract. They include *Saccharomyces boulardii* yeast and lactic acid bacteria, such as *Lactobacillus* and *Bifidobacterium* species [190].

Probiotics offer benefits through several mechanisms, including lowering intestinal pH, reducing colonization and invasion by pathogenic organisms, and influencing the host immune response [190]. However, benefits observed with one species or strain cannot automatically be applied to others. The strongest clinical evidence is for their use in treating acute diarrhea, especially caused by rotavirus [190].

In a very recent study from 2024, it was found that administering probiotics (*Lactobacillus rhamnosus GG* and *Bifidobacterium longum*) worsened gut-derived *P. aeruginosa* sepsis in chemotherapy-treated mice. Instead of offering protection, probiotics increased inflammation, weakened the gut barrier and promoted bacterial translocation to the liver, spleen, and blood. These findings revealed that probiotics may be harmful in immunocompromised settings, and their use in patients undergoing chemotherapy complicated by *P. aeruginosa* infection should be approached with caution [191].

A different study focused on the protective properties of probiotics in case of a human eye infection. During the research it was revealed that *Lactobacillus reuteri* and *Bifidobacterium longum* subsp. *infantis* can protect human corneal epithelial cells from a *P. aeruginosa* infection. Pretreatment with these probiotics preserved tight junction integrity, increased mucin production, improved cell viability, and reduced inflammation and nitrosative stress. Overall, these results suggested that probiotics could serve as a preventive strategy against *P. aeruginosa*-induced corneal infections. However, despite these interesting initial findings, researchers advise further studies to better understand probiotics efficacy and mechanisms in the process of corneal infection [192].

### 5.12. Lactoferrin and Hypothiocyanite Combination Therapy

Hypothiocyanite (OSCN^−^), a reactive antimicrobial compound that is part of the innate host immune defense, is naturally produced in the airways via CFTR-dependent transport and enzymatic reactions [193]. Its production is impaired in CF patients due to CFTR dysfunction, that reduces thiocyanate (SCN^−^) transport to the airway surface, limiting its conversion to OSCN^−^ by lactoperoxidase [193]. The lack of this compound weakens hosts’ airway’s natural antimicrobial defenses, contributing to lower ability to fight bacterial infections, particularly against *P. aeruginosa* [193]. Therefore, transporting supplementary OSCN^−^ into the CF lung has been shown to help control *P. aeruginosa* colonization [193].

As a result, an inhalation therapy combining lactoferrin (potent antimicrobial, anti-inflammatory and iron-chelator) with hypothiocyanite was proposed and received orphan drug status from both the European Medicines Agency (EMA) and the U.S. Food and Drug Administration (FDA) in 2009 [194]. Other researchers investigated the combination of OSCN^−^ and lactoferrin with antibiotics tobramycin and aztreonam. It was found that while OSCN^−^ and lactoferrin reduced the formation of new *P. aeruginosa* biofilms, they had no effect on already established biofilms. However, this combination enhanced effectiveness of tobramycin and aztreonam, thus reducing existing *P. aeruginosa* biofilms [195].

Given the previously positive results, a renewed approach, which potentially includes clinical study, could help to revive interest in this combination therapy. The results of the reviewed studies were promising after all and both EMA and FDA awarded the novel method with the orphan drug status, which shows a certain degree of confidence in the approach.

Despite promising antimicrobial and antibiofilm properties, the therapeutic combination of lactoferrin and hypothiocyanite (OSCN^−^) faces several important limitations. First, existing evidence indicates that while the formulation effectively inhibits the formation of new *P. aeruginosa* biofilms, it demonstrates minimal activity against mature established biofilms, necessitating co-administration with conventional antibiotics to achieve meaningful biofilm reduction [195]. Furthermore, optimal dosing parameters for both lactoferrin and OSCN^−^ remain insufficiently defined, and current studies suggest that bacterial regrowth may occur after treatment discontinuation, raising concerns about the durability of its therapeutic effect [195]. The combination’s broader impact on airway microbial communities also remains unclear, as its non-selective antimicrobial activity may alter host-microbiota interactions in ways not yet fully characterized. Finally, clinical evidence is limited: although the therapy has received orphan drug designation, comprehensive clinical trials validating its safety, pharmacodynamics, and efficacy in CF patients are still lacking, representing a major barrier to its translation into routine therapy [195].

### 5.13. Electrochemical Scaffolds

A recently proposed strategy for eliminating bacterial contaminations involves using electrochemical scaffolds to generate a low but consistent concentration of H_2_O_2_. Electrochemical scaffolds are electrically conductive or electrochemically active materials that, when polarized with an external potential, electrochemically generate localized antimicrobial chemical species (such as hydrogen peroxide or hypochlorous acid) at their surface, which can disrupt bacterial biofilms and reduce microbial viability without relying on conventional antibiotics [196,197,198,199]. One study showed that H_2_O_2_ produced, for example, by a steel surface, significantly reduced *P. aeruginosa* biofilm formation [196]. In a separate study, it was demonstrated that the electrochemical scaffold (e-scaffold) induced intracellular formation of hydroxyl free radicals and improved membrane permeability in biofilms cells [197]. Thus, it enhances antibiotic susceptibility and eradicates “persister” cells [197]. These findings demonstrate a promising potential of the e-scaffold in treating persistent biofilm infections.

Recent experimental studies have utilized this new treatment mechanism and focused on addressing topical wound infections with antibiotic-resistant bacterial pathogens, particularly *P. aeruginosa*. In a study by Fleming et al. from early 2024, a novel electrochemical bandage (e-bandage) delivering low levels of hypochlorous acid (HOCl^−^) was tested against *P. aeruginosa* biofilms [198]. In this experiment, 5 mm skin wounds were created on the dorsum of mice and infected with 10^6^ colony-forming units (CFU) of *P. aeruginosa*. Biofilms developed over 2 days, after which e-bandages were applied to the wound beds and covered with tegaderm (transparent adhesive film dressing by 3M). Mice were treated with either a tegaderm-only (control) dressing, a non-polarized e-bandage (no HOCl^−^ production), or a polarized e-bandage (producing HOCl^−^ via a potentiostat), and with or without systemic amikacin (aminoglycoside antibiotic) [198].

After 48 h, the wounds were harvested for bacterial quantification. The results showed that the HOCl^−^ producing e-bandage effectively reduced *P. aeruginosa* in wound biofilms. However, neither the polarized nor non-polarized e-bandage treatments affected wound closure. Systemic amikacin also had no impact on wound healing or purulence. This suggests that the e-bandage was effective at fighting the bacterial infection without disrupting the healing process.

The researchers concluded that this method could become a capable antibiotic-free treatment approach for topical infections caused by *P. aeruginosa* and possibly other MR-pathogens [198].

In the same year, Fleming et al. revisited the topic of the electrochemical bandage (e-bandage) and published an updated study. In this work, the authors highlighted that, unlike traditional therapies, which may have limited effectiveness against biofilms and antimicrobial-resistant organisms, the e-bandage offers a potent, standalone solution. It does not contribute to further resistance and does not require adjunctive antibiotic therapy. The study also demonstrated the e-bandage’s ability to combat polymicrobial infections caused by antimicrobial-resistant clinical isolates of *S. aureus* and *P. aeruginosa*. The researchers emphasized the potential of this HOCl^−^ producing e-bandage as an effective tool for managing complex wound infections, noting its ability to reduce pathogen load while minimizing tissue toxicity and eliminating the need for systemic antibiotics [199].

Despite the promising antimicrobial and antibiofilm activity of HOCl^−^ producing electrochemical bandages, several challenges limit their translation into routine clinical use. Most studies to date have been conducted in in vitro biofilm models or animal wound models, which do not fully replicate the complexity of human wounds, including tissue architecture, immune responses, and variable wound exudate, potentially limiting the predictive value of preclinical findings. Moreover, comprehensive in vivo studies in larger animal models or humans are still lacking, leaving uncertainty about how factors such as host enzymes, biofilm matrix barriers and wound heterogeneity influence HOCl^−^ generation and efficacy. Physical factors in real wounds, such as scab formation or dense biofilm matrices, may impede the penetration of electrochemically active conditions. While low concentrations of HOCl^−^ are intended to minimize tissue toxicity, precise safety thresholds across diverse wound tissues remain undefined, particularly during prolonged or repeated applications. Additionally, the novel mechanism of action and device requirements, including wearable electrodes and controlled electrochemical polarization, pose regulatory and standardization challenges, as these systems do not fit neatly into existing pathways for antimicrobial or wound care devices. Finally, broader translational hurdles common to anti-biofilm therapies, such as the limited predictive power of preclinical models and the absence of standardized efficacy testing, further slow clinical adoption. Collectively, these factors highlight the need for further research to validate safety, optimize delivery, and establish standardized protocols before e-bandages can be widely implemented in routine wound care.

Table 1 below provides an overview of the novel therapeutic approaches currently being explored against MRPA.

## 6. Conclusions

*P. aeruginosa* remains a formidable pathogen due to its rapid development of multidrug-resistance, biofilm formation, and diverse virulence factors. Key barriers to translating novel therapies into clinical practice include incomplete understanding of mechanisms in complex in vivo environments, strain-specific efficacy, potential cytotoxicity, stability issues, and the lack of extensive clinical trials.

Among emerging strategies, the most realistic near-term approaches include iron chelation, quorum-sensing inhibitors, bacteriophage therapy, electrochemical scaffolds, lectin and efflux pump inhibitors, and targeted use of newer antibiotics for difficult-to-treat resistant strains. These methods show promising in vitro and limited in vivo results, though further research is required to optimize safety, dosing and delivery.

Ultimately, the take-home message is that while antibiotics remain the cornerstone of therapy, careful stewardship combined with the development of adjunctive or alternative approaches is essential. Translating promising laboratory findings into clinically effective treatments will require rigorous preclinical testing, in vivo validation, and well-designed clinical trials to address persistent *P. aeruginosa* infections.

## Figures and Tables

**Table 1:** Emerging therapeutic strategies against MRPA.

Therapeutic Strategy/Agent	Mechanisms Targeted	Evidence Level	Key Limitation	References
Quorum-sensing inhibitors (QSIs) and quorum quenching (QQ)	QS receptor binding, signal molecule production, bacterial communication; biofilm regulation; virulence factor expression	In vitro and some in vivo studies	Poor delivery and bioavailability; difficulty achieving therapeutic concentrations at infection sites; potential effects on human cells and disruption of normal microbiota; risk of development of QSI-resistant pathogens over time; need for optimized formulations and precise delivery strategies	[3,10,18,55,56,65,66,67,68,69,70,71,72,73,74,75,76,77,78,79,80,81,82,83,84,85]
Iron chelators	Siderophore binding; Fe^3+^ mimicry; inhibition of pyoverdine/pyochelin production; iron sequestration; disruption of bacterial metabolism and biofilm formation.	In vitro and in vivo	Cytotoxicity, systemic adverse effects; strain-dependent antimicrobial activity; poor stability, limited tissue penetration, rapid inactivation in vivo; compensatory bacterial responses; reduced effectiveness in biofilms and polymicrobial environments; need for optimized formulations, targeted delivery and robust in vivo validation	[86,87,88,89,90,91,92,93,94,95,96,97]
Lectin inhibitors (e.g., β-phenylgalactosyl peptide dendrimers)	Inhibits adhesion to abiotic surfaces and epithelial cells; interferes with lectin-mediated biofilm formation; reduces host inflammatory response	in vitro cell line studies	Design complexity and large multivalent structures leading to unfavorable pharmacokinetics; potential off-target effects and unknown medical concerns; difficult and costly synthesis; lack of robust in vivo and clinical data on safety and efficacy; strain-dependent efficacy	[98,99,100,101,102,103,104,105,106,107]
Efflux pump inhibitors	RND family efflux pumps (MexAB-OprM, MexXY-OprM)	In vitro	Loss of efficacy in complex biological systems; large chemically complex structures leading to poor pharmacokinetics, low solubility, limited tissue penetration; toxicity and off-target effects; regulatory hurdles and high development costs; resistance evolution	[108,109,110,111,112,113,114,115,116,117,118]
Traditional, natural and phytochemical medications against *P. aeruginosa*	Cell membrane disruption	In vitro	Safety concerns with high doses pr prolonged use; lack of robust human clinical data; variability in plant sources and preparation methods, causing inconsistent efficacy; complex mixtures complicating dose optimization and safety assessment; limited in vivo and toxicological data; poor bioavailability	[119,120,121,122,123,124,125,126,127,128,129,130,131]
Preventive immunization targeting antigens	Bacterial antigens inducing adaptive immune response	In vivo	Limited understanding of human immune responses; dependence on murine models for data; need for multifaceted immune activation; translation to clinical vaccination trials remains uncertain.	[132,133,134,135,136,137,138,139]
Nanoparticles	Membrane penetration, ROS induction, enzymatic inhibition, DNA/RNA interaction, protein synthesis inhibition, drug delivery to biofilm cells, virulence suppression (pyocyanin, motility, EPS)	In vitro, early preclinical, and some animal studies	Potential toxicity, organ-system effects unknown, controlled release challenges, regulatory hurdles	[140,141,142,143,144,145,146,147,148,149,150,151,152,153,154,155]
Bacteriophage therapy	Bacterial lysis, biofilm penetration, quorum sensing interference, alginate degradation, virulence factor suppression, host-specific targeting	In vitro, in vivo; murine models; some preclinical personalized phage cocktails	Safety concerns (phage clearance, impurities, production issues), poor stability, limited mechanistic understanding of phage-host interactions; bacterial resistance to phages; immune system neutralization of phages; need for extensive clinical trials	[156,157,158,159,160,161,162,163,164,165,166,167,168,169]
Antimicrobial peptides (AMPs)	Membrane disruption via pore formation, outer membrane destabilization, biofilm inhibition, quorum sensing suppression, enhanced antibiotic uptake, virulence factor suppression	In vitro, in vivo	Hemolytic activity, proteolytic degradation, salt/serum/pH sensitivity, high production cost, limited clinical data, potential rapid degradation, stability/toxicity concerns.	[170,171,172,173,174,175,176,177,178,179,180,181]
Novel antibiotics	Cell wall synthesis inhibition, protein synthesis inhibition, DNA replication inhibition, LPS transport inhibition, β-lactamase inhibition, broad-spectrum antimicrobial activity	In vitro, in vivo;	Most are modifications of existing drug classes, limited activity against metallo-β-lactamase producers, some carbapenems ineffective against *P. aeruginosa,* potential nephrotoxicity, ototoxicity, resistance development, limited long-term effectiveness.	[182,183,184,185,186,187,188,189]
Oral probiotics	Modulation of mucosal immunity; inhibition of pathogen adhesion, growth, cytotoxicity and biofilm formation	Limited preclinical data; no robust evidence for clinical benefit	Potential harm in immunocompromised host; strain-specific effects; risk of systemic infection; interaction with antibiotics	[190,191,192]
Lactoferrin and hypothiocyanite combination therapy	Restore innate airway antimicrobial defense, inhibit new biofilm formation, enhance antibiotic efficacy	Preclinical and ex vivo studies; EMA/FDA orphan drug status	Limited activity against mature biofilm; need for co-administration with antibiotics; undefined optimal dosing; potential bacterial regrowth after treatment; unclear impact on airway microbiota; limited clinical evidence and lack of trials	[193,194,195]
Electrochemical scaffolds	Generates reactive oxygen species (ROS) such as H_2_O_2_ and HOCl; increases biofilm cell membrane permeability; eradicates persister cells; enhances antibiotic susceptibility.	Preclinical, in vivo murine wound infection models	Limited predictive value of preclinical models; lack of comprehensive in vivo studies; physical barriers in real wounds; undefined safety thresholds, regulatory and standardization challenges, translational hurdles common to anti-biofilm therapies	[196,197,198,199]

## Data Availability

No new data were created or analyzed in this study.

## Abbreviations

The following abbreviations are used in this manuscript:

CF	Cystic fibrosis
COPD	Chronic obstructive pulmonary disease
DTR	Difficult-to-treat resistant
IDSA	The Infectious Diseases Society of America
ESCMID	European Society of Clinical Microbiology and Infectious Diseases
HGT	Horizontal gene transfer
LPS	Lipopolysaccharides
MRPA	Multidrug-resistant *P. aeruginosa*
QSI	Quorum sensing inhibitors
QQ	Quorum quenching
EPI	Efflux pump inhibitors
PAβN	Phenylarginine-β-naphthylamide
RND	Resistance-Nodulation-Division
APDI	Antimicrobial photodynamic inactivation
MB	Methylene blue
ROS	Reactive oxygen species
MIC	Minimum inhibitory concentration
OMP	Outer membrane protein
NP	Nanoparticle
CRISPR	Clustered Regularly Interspaced Short Palindromic Repeats
AMP	Antimicrobial peptide
OSCN	Hypothiocyanite
CFTR	Cystic fibrosis transmembrane conductance regulator
EMA	European Medicines Agency
FDA	U.S. Food and Drug Administration
HOCl	Hypochlorous acid
